# Cell Line Characteristics Predict Subsequent Resistance to Androgen Receptor-Targeted Agents (ARTA) in Preclinical Models of Prostate Cancer

**DOI:** 10.3389/fonc.2022.877613

**Published:** 2022-06-13

**Authors:** Jan Matthijs Moll, Wilma J. Teubel, Sigrun E. Erkens, Ashraf Jozefzoon-Agai, Natasja F. Dits, Angelique van Rijswijk, Guido W. Jenster, Wytske M. van Weerden

**Affiliations:** Department of Urology, Erasmus Medical Centre (MC), Rotterdam, Netherlands

**Keywords:** CRPC, ARTA, PC346C, VCaP, DuCaP, LAPC4

## Abstract

Treatment of prostate cancer (PCa) has changed considerably in the last decade due to the introduction of novel androgen receptor (AR)-targeted agents (ARTAs) for patients progressing on androgen deprivation therapy (ADT). Preclinical research however still relies heavily on AR-negative cell line models. In order to investigate potential differences in castration-resistant PCa (CRPC) growth, we set out to create a comprehensive panel of ARTA-progressive models from 4 androgen-responsive AR wild-type PCa cell lines and analyzed its androgen response as opposed to its ADT-progressive counterparts. Parallel cultures of VCaP, DuCaP, PC346C, and LAPC4 were established in their respective culture media with steroid-stripped fetal calf serum (FCS) [dextran-coated charcoal-stripped FCS (DCC)] without androgen (ADT) or in DCC plus 1 μM of the ARTAs bicalutamide, OH-flutamide, or RD162 (an enzalutamide/apalutamide analog). Cell growth was monitored and compared to those of parental cell lines. Short-term androgen response was measured using cell proliferation 3-(4,5-dimethylthiazol-2-yl)-2,5-diphenyltetrazolium bromide (MTT) assay. qRT-PCR was performed to assess the mRNA expression of markers for AR signaling, steroidogenesis, glucocorticoid receptor (GR) signaling, epithelial-mesenchymal transition (EMT), and WNT signaling. Out of 35 parallel cultures per cell line, a total of 24, 15, 34, and 16 CRPC sublines emerged for VCaP, DuCaP, PC346C, and LAPC4, respectively. The addition of bicalutamide or OH-flutamide significantly increased CRPC growth compared to ADT or RD162. VCaP, DuCaP, and PC346C CRPC clones retained an AR-responsive phenotype. The expression of AR and subsequent androgen response were completely lost in all LAPC4 CRPC lines. Markers for EMT and WNT signaling were found to be elevated in the resilient PC346C model and CRPC derivatives of VCaP, DuCaP, and LAPC4. Although the resistant phenotype is pluriform between models, it seems consistent within models, regardless of type of ARTA. These data suggest that the progression to and the phenotype of the CRPC state might already be determined early in carcinogenesis.

## Introduction

Due to the dependency of normal prostate (1) and prostate cancer (PCa) tissue (2) on androgen receptor signaling for development and proliferation, the main treatment for metastatic PCa is androgen deprivation therapy (ADT) (3). However, the development of resistance to androgen ablation is inevitable, with all metastatic hormone-sensitive PCa (HSPC) patients developing castration-resistant PCa (CRPC), indicated by a rising serum prostate-specific antigen (PSA) or progressive disease despite castrate levels of serum androgens.

Persisting AR signaling has long since been recognized as a resistance mechanism to ADT. However, maximal androgen blockade (MAB) by addition of the antiandrogens bicalutamide (BIC) (4) or OH-flutamide (FLU) (5) to ADT has not shown a survival benefit over ADT alone. In the last decade, novel androgen receptor-targeted agents (ARTAs) have demonstrated efficacy in the treatment of CRPC and have quickly become standard of care for advanced PCa patients.

Abiraterone, an androgen synthesis inhibitor with antiandrogen properties, targets the AR by further suppression of available androgens in the tumor microenvironment (6) and has demonstrated clinical benefit in both CRPC (7, 8) and HSPC (9). Inhibiting AR *via* direct antagonism (10), adding enzalutamide to ADT has demonstrated clinical benefit in the treatment of metastatic PCa, both in HSPC (11, 12) and CRPC (13–15). Addition of apalutamide, an AR antagonist with similar potency as enzalutamide (16), equally demonstrated clinical benefit in CRPC (17) and HSPC (18). However, resistance to ARTA and subsequent CRPC ultimately arise, with the majority of patients requiring further systemic treatment with cytotoxic agents (19).

The traditional *in vitro* models considered representative of CRPC are AR-negative (PC3, DU145, and MDA PCa1), a phenomenon that remains rare in CRPC. Traditional HSPC models like LNCaP, MDA PCa2, VCaP, DuCaP, and LAPC4 are derived from PCa metastases after hormone treatment. Nevertheless, these cell lines need androgens for their optimal growth and survival and therefore are considered androgen-responsive (20–24). *In vitro* PCa cell lines from primary tumors such as PC346C and 22Rv1 are less common (25–27). Likely selected by the hormone treatment, 22Rv1, LNCaP, and MDA PCa2 harbor AR mutations (23, 28), while the other cell lines are AR wild type (wt) but often overexpress the AR. The PC346C cell line has been established from a xenograft from a transurethral resection of the prostate after a 4-week cyproterone acetate treatment (25). In order to establish relevant models of PC346C CRPC, we previously generated a set of CRPC clones and found that the hormonal response varied greatly between the CRPC sublines. Three different CRPC phenotypes were identified with AR amplification (PC346FLU1), AR mutation (PC346FLU2), and AR bypass (PC346DCC) (29). From these experiments, we learned that one needs to independently establish many different replicate clones to represent this broad spectrum of adaptability.

To further investigate if the AR is the dominant mechanism of CRPC in other AR wt cell lines, we set out to generate an extensive panel (5–10 replicates per condition) of CRPC clones from 4 distinct AR wt PCa cell lines (VCaP, DuCaP, PC346C, and LAPC4) by growing them in androgen-depleted medium alone (ADT) or with ARTA BIC, FLU, or RD162, an antiandrogen with a similar molecular structure and antiandrogenic efficacy as enzalutamide and apalutamide (10). Once CRPC lines were established, they were further characterized on androgen responsiveness and expression of AR, AR target genes, and a panel of genes that have previously been described as drivers of castration resistance and resistance to second-line hormonal agents in PCa. These genes include the ligand-independent AR variant V7, the steroidogenic enzymes CYP17A1, AKR1C3, and SRD5A1, the glucocorticoid receptor (GR) and its target gene SGK1, the MYC oncogene, the stem cell factor IL6, EMT markers ZEB-1 and SNAI1, and wnt-signaling genes WNT5A and WNT7B (**Table 1**).

**Table 1:** CRPC-associated genes selected for qPCR analysis.

Mechanism	GENE	Diffential expression					Function	Ref
		*Between original cell lines*	*Between CRPC lines per original cell line*					
		PAR	VCAP	DUCAP	PC346C	LAPC4		
AR signaling	AR	**0.002**	0.894	**0.001**	**<0.001**	0.195	*Androgen receptor, main growth factor receptor in prostate cancer, target of androgen-ablative therapy*	(2)
	PSA	0.086	0.560	0.939	**0.011**	**0.001**	*Target gene of AR, prostate cancer biomarker.*	
	FKBP5	**0.021**	**0.008**	**0.026**	**0.010**	**<0.001**	*Target gene of AR*	
Ligand-independent AR	AR-V7	**0.001**	**0.003**	0.102	**<0.001**	**0.013**	*Ligand-independent AR signaling*	(30)
Steroid biosynthesis	CYP17A1	0.138	**<0.001**	**0.029**	0.581	0.424	*Conversion of pregnenolone to 17OH-pregnenolone and DHEA, target of abiraterone*	(31)
	AKR1C3	**0.002**	**0.007**	**0.002**	0.154	0.073	*Conversion of DHEA and androstenedione into testosterone and DHT*	
	SRD5A1	**0.003**	**0.018**	0.314	0.104	0.714	*Conversion of testosterone into more potent DHT*	
GR signaling	GR	0.120	0.150	0.523	**<0.001**	**0.012**	*Glucocorticoid receptor, potential alternative growth factor receptor*	(32, 33)
	SGK1	**0.047**	0.543	0.916	0.249	0.416	*Target gene of GR*	
Alternative growth factor	MYC	0.069	0.145	0.186	0.054	0.495	*Oncogene*	(34, 35)
	IL6	0.457	na	na	0.400	0.321	*Stem-cell signaling, autocrine growth factor*	(36)
EMT	ZEB-1	0.636	0.206	0.189	0.724	**0.001**	*Direct drivers of EMT, associated with castration and chemoresistance in prostate cancer*	(37)
	SNAI1	**0.007**	**<0.001**	0.065	**0.001**	**0.006**	
Wnt signaling	WNT5A	**0.019**	na	0.375	**0.003**	0.229	*Stem cell signaling, CRPC, resistance to ADT, abiraterone and enzalutamide*	(38, 39)
	WNT7B	0.457	0.381	0.544	0.160	**0.039**	

Bold numbers indicate p<0.05 on a one-way ANOVA.

AR, Androgen Receptor; GR, Glucocortioid Receptor; EMT, Epithelial-Mesenchymal transition; PSA, prostate specific antigen; FKBP5, FK506 binding protein 5; AR-V7, Androgen Receptor splice variant 7; CYP17A1, Cytochrome P450 17A1; AKR1C3, Aldo-keto reductase family 1 member C3; SRD5A1, Steroid 5 Alpha-Reductase 1; GR, glucocorticoid receptor; SGK1, Serum/Glucocorticoid Regulated Kinase 1; MYC, MYC Proto-Oncogene; IL6, interleukin 6; ZEB-1, Zinc Finger E-Box Binding Homeobox 1; SNAI1, Snail Family Transcriptional Repressor 1; WNT5A, Wnt family member 5A; WNT7B, Wnt family member 7B; PAR, parental; DHEA, Dehydroepiandrosterone; DHT, dihydrotestosterone; CRPC, Castration-resistant prostate cancer; ADT, androgen deprivation therapy; na, not applicable.

## Materials and Methods

### Original Cell Lines

PC346C (p25) was generated in our lab as described previously (25, 26) and maintained in prostate growth medium (PGM) consisting of DMEM-F12, supplemented with 2% FCS (both from Lonza, Breda, Netherlands), 1% insulin-transferrin-selenium (Gibco), 100 ng/ml fibronectin (Harbor Bio-Products, Tebu-bio, Netherlands), 20 μg/ml fetuin (ICN Biomedicals, Zoetermeer, Netherlands), 0.01% BSA, 10 ng/ml epidermal growth factor, 50 ng/ml cholera toxin, 0.1 mM phosphoethanolamine, 0.6 ng/ml triiodothyronine, 500 ng/ml dexamethasone (all from Sigma-Aldrich), 0.1 nM R1881 (Sigma), and penicillin/streptomycin antibiotics (100 U/ml penicillin, 100 μg/ml streptomycin; Cambrex BioWhittaker). VCaP (p30) (20) (kindly provided by Dr. K.J. Pienta, Baltimore, MD, USA) and DuCaP (p52) (21) (kindly provided by Dr. J.A. Schalken, Nijmegen, Netherlands) were maintained in RPMI1640 (Lonza), supplemented with 10% FCS and penicillin/streptomycin antibiotics. LAPC4 (p49) (22) was kindly provided by Organon (Oss, Netherlands) and maintained in Iscove's Modified Dulbecco's Medium (IMDM) (Lonza), supplemented with 7.5% FCS and 10 nM R1881. Cell line authentication was performed by short tandem repeat analysis by the Promega PowerPlex 16 kit.

### Selection Medium

The ADT condition consisted of each parental cell line’s respective medium, substituting FCS with dextran-coated charcoal-stripped FCS (DCC) and removal of R1881. For ARTA conditions, BIC (kindly provided by Organon), FLU (Shering Plough Research Institute), or RD162 (Axxon Medchem, Netherlands) was added to the medium at a final concentration of 1 μM. Cell culture medium was refreshed twice per week, and at 80%, confluency cells were trypsinized and passaged. Once time between passaging approximated that of parental cell lines grown in standard androgen-supplemented medium, clones were considered resistant.

### Proliferation Assays

To test androgen responsiveness, cells were plated in steroid-stripped medium at 10,000 cells per well in 100-μl medium in 96-well culture plates with 8 replicates per condition. After overnight attachment, medium with R1881 and BIC, FLU, or RD162 was added with ethanol (for R1881) and dimethyl sulfoxide (DMSO) (for ARTA) as vehicle controls. Based on preliminary androgen response curves, we selected 0, 10^-11^, 10^-10^, and 10^-9^ M R1881 for VCaP, DuCaP, and PC346C as androgen concentration range for proliferation assays. For LAPC4, we selected 0, 10^-10^, 10^-9^, and 10^-8^ M. After a 9-day incubation period at 37°C and 5% O_2_, cell numbers were estimated using the 3-(4,5-dimethylthiazol-2-yl)-2,5-diphenyltetrazolium bromide (MTT) assay as described previously (40).

### Androgen Response Analysis

All proliferation assay results were summarized using the average MTT signal intensity for each condition relative to the MTT signal intensity at day 0. Basal growth was assessed in the absence of steroid and with vehicle control. Growth induction by increasing concentrations of R1881 was assessed relative to vehicle control. The effect of ARTA was determined relative to vehicle control at the specified amount of R1881. To group growth responses, values were log_2_ transformed, clustered using Cluster (version 3.0) (41) using complete linkage based on Euclidian distance, and visualized by Java TreeView (version 1.16r4) (42).

### Quantitative Real-Time PCR

For qPCR, cells harvested at passaging were stored as cell pellets at -80°C until further processing. RNA was isolated using RNA-Bee (TEL-TEST, Inc., Friendswood, USA). Reverse transcriptase and qPCR runs were performed as described previously (43) using an ABI Prism 7900 Sequence Detection System under standard conditions. cDNA (20 ng) was amplified in SYBR Green PCR Master Mix (Applied Biosystems, Foster City, USA) or TaqMan Universal Master Mix (Applied Biosystems). PCR efficiency was verified by cDNA dilution curves and exceeding 90% for all assays. Gene expression was calculated as fold expression over housekeeping genes glyceraldehyde 3-phosphate dehydrogenase (GAPDH) and porphobilinogen deaminase (PBGD).

### mRNA Expression Analysis

Relative gene expression levels were expressed as ΔCt relative to the geometric mean of housekeeping genes GAPD and PBGD. GraphPad Prism (version 9) was used to group and visualize -ΔCt values. For cluster analyses, -ΔCt values were median centered per gene and clustered using Cluster using complete linkage with Spearman Rank Correlation (version 3.0) and visualized by Java TreeView (version 1.16r4). For statistical analyses and clustering of samples, undetectable gene expression (Ct >40) was normalized to a –ΔCt of -20.

### Statistical Analysis

All statistical analyses were carried out using SPSS version 26 (IBM, Armonk, NY, USA). The χ^2^ test was used to test the null hypothesis that overall take rates between cell lines, between ADT and ARTA, and between ADT and ARTA within each cell line were equal. For the proliferation assay analysis, a regular two-way ANOVA was used to test the null hypothesis that fold growth rates between parental and CRPC cells and between hormonal/ARTA conditions were equal. For comparison of mRNA expression levels between cell lines and culture conditions, the ΔCt values were used as input to maintain a normal distribution on a log2 scale, using a regular one-way ANOVA with a Tukey-corrected *post-hoc* test to test the null hypothesis that these were equal between parental cell lines and/or ADT and specified ARTA. For all tests, a p < 0.05 was considered statistically significant. To assess at what time CRPC cell growth had stabilized, 5-knot smooth splines were fitted through passage times, defined as time in days until next passage, on the y-axis and passage number on the x-axis using GraphPad Prism (version 9).

## Results

### Primary Cell Line and AR-Targeted Agents (ARTA) Determine the Emergence of Castration-Resistant Prostate Cancer (CRPC)

**Figure 1:**
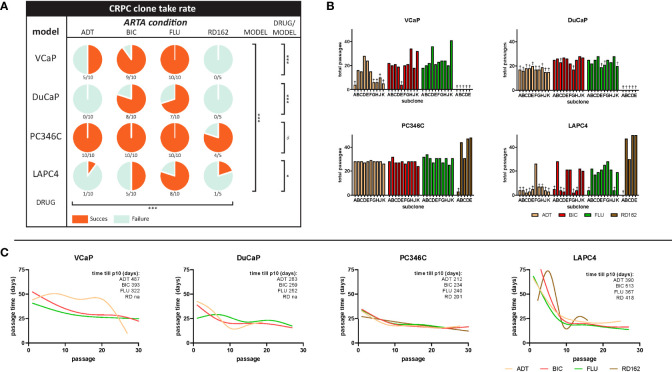
Success rates and cell culture dynamics of CRPC sublines. **(A)** Survival rate of CRPC sublines per cell line and hormonal condition. Coral sectors represent a fraction of established clones relative to attempts. Overall effect of type of ARTA (ADT vs. BIC vs. FLU vs. RD162), p < 0.0001. Overall primary cell line effect (VCaP vs. DuCaP vs. PC346C vs. LAPC4), p < 0.0001. Drug effect per cell line: VCaP, p = 0.0002; DuCaP, p = 0.0002; PC346C, p = 0.10; LAPC4, p = 0.01 (χ^2^ test). **(B)** Total passages in culture per clone until the end of the experiment or failure. † = failure to proliferate beyond the noted passage. **(C)** Smooth spline curves of average passage time at subsequent passages stratified per cell line and ARTA condition. Overall, passage time did not stabilize before the 10th passage. Top right numbers indicate the mean time in days until the 10th passage per ARTA condition for each cell line group. “ns” is “not significant”; “*” is “* p<0.05”; “***” is “*** p<0.001”.

PCa cell lines VCaP, DuCaP, PC346C, and LAPC4 were long-term cultured in 5–10 independent replicates in androgen-depleted medium alone (ADT) or with ARTA BIC, FLU, or RD162. The emergence of CRPC sublines (**Figure 1A**) varied substantially between cell line origin and per selection condition. Overall, PC346C was more prone to generate a CRPC cell line with an overall success rate of 97% vs. 69%, 42%, and 42% for VCaP, DuCaP, and LAPC4, respectively (p < 0.0001).

Strikingly, the addition of FLU or BIC to the medium at 1 μM significantly increased the success rate of CRPC subline generation compared to castrate medium (ADT) alone (p < 0.0001). However, this effect was cell line-dependent, with PC346C showing no preference for any selective pressure, while VCaP, DuCaP, and LAPC4 showed enhanced CRPC survival in the presence of BIC or flutamide compared to either ADT or the more potent AR antagonist RD162 (**Figure 1A**).

The median passage number (range) for successful establishment of CRPC sublines with similar growth rates as the parental line was 21 (14–41) for VCaP, 24 (21–27) for DuCaP, 27 (23–47) for PC346C, and 20 (18–27) for LAPC4 (**Figure 1B**). Median total selection time was 635, 587, 508, and 589 days for VCaP, DuCaP, PC346C, and LAPC4, respectively. Median number (range) of passages to definitive failure, defined as the inability to grow after passaging, was 3 (0–9) for VCaP, 16 (0–22) for DuCaP, 2 for PC346C, and 3 (0–6) for LAPC4. Median (range) days to definitive failure were 208 (131–635) for VCaP, 404 (390–440) for DuCaP, 77 for PC346C, and 215 (56–247) for LAPC4. Of note, DuCaP sublines took significantly longer to demonstrate failure due to contamination with murine stromal cells, which is in line with a previous report (41). This made it harder to identify which flasks no longer contained viable epithelial cells (**Supplementary Figure S1**).

To test at what passage of CRPC subline growth rate stabilized, indicative of full adaptation to its selection medium, we plotted the average time between passages of the selection cultures against the passage number. Smooth splines fitted through these data revealed that growth rates did not stabilize before the 10th passage from initiation of treatment (**Figure 1C**; **Supplementary Figure S2**).

### Primary Cell Line Dictates the Castration-Resistant Phenotype

**Figure 2:**
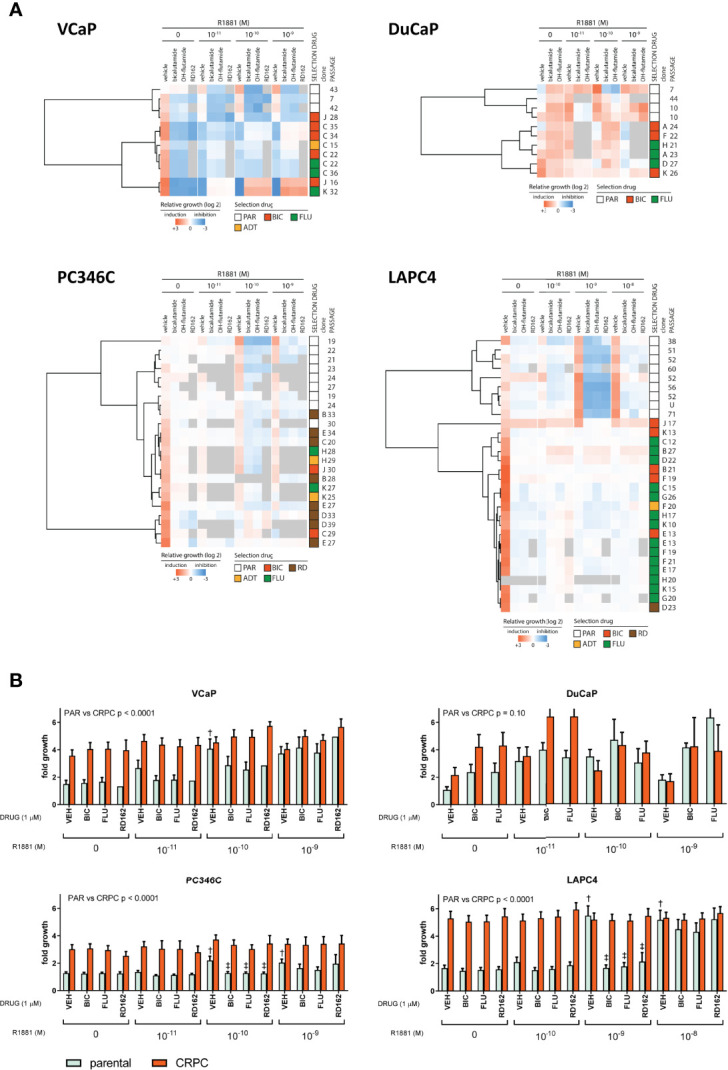
Androgen growth response and antiandrogen potency in CRPC clones. **(A)** Heatmap of growth response of individually tested clones. First column represents fold growth relative to day 0. Subsequent vehicle columns represent growth induction (red) or inhibition (blue) relative to no R1881/vehicle. Each column with antiandrogen (bicalutamide, OH-flutamide, RD162) represents a normalized response to the corresponding vehicle control at 1 μM ARTA in the presence of different concentrations of R1881. Gray = missing data. **(B)** Average proliferation for parental and CRPC clones. Average fold growth relative to day 0 in the MTT assay for parental cells (mint bars) and all CRPC cells combined (coral bars). The p value represents parental vs. CRPC in two-way ANOVA. ^†^p < 0.05 vs. no R1881/vehicle. ^‡^p < 0.05 vs. vehicle control at a given amount of R1881.

We subsequently tested a random set of the generated CRPC cell lines for its response to ADT and ARTA. Clustering proliferation assay data revealed reproducible and cell line-specific differences in androgen response. For VCaP, DuCaP, LAPC4, and to a lesser extent PC346C, the greatest distance in AR growth response patterns was observed between parental and CRPC sublines (**Figure 2A**). CRPC sublines in all 4 models clustered together, with no differences in androgen response between sublines that became resistant to either ADT or ARTA with BIC, FLU, or RD162. As resistant sublines from the same cell line revealed a similar androgen response, we subsequently combined proliferation assay results of the parental and CRPC cell lines for an overall response visualization (**Figure 2B**).

Apart from all CRPC sublines showing higher proliferation rates in castrate conditions, we found marked differences in AR response for CRPC sublines from each model. The growth of VCaP CRPC sublines was stimulated at lower levels of R1881 compared to parental cells (10^-11^ vs. 10^-10^ M) and inhibited at 10^-9^ M R1881, with further growth induction with AR antagonists at 1 µM at 10^-10^ R1881 and up, implying hypersensitivity to the AR stimulus. Parental and CRPC DuCaP sublines showed growth induction with 1 µM ARTA with an optimal growth stimulus at 10^-11^ M. Interestingly, higher levels of R1881 (10^-9^ M) that were growth stimulatory for parental DuCaP showed growth inhibition in the CRPC clones. This demonstrates an increased AR hypersensitivity in CRPC sublines. In PC346C, androgens continued to induce cell growth in CRPC sublines with a similar optimum concentration of 10^-10^ M R1881 as observed in the parental lines. Incubation with ARTA reduced androgen-induced cell growth both in parental and CRPC sublines. However, the increased basal proliferation of CRPC sublines (compared to parental samples) in castrate conditions was virtually unaffected by ARTA, implying that castration-resistant growth was independent of AR.

LAPC4 clones revealed the largest difference between parental and CRPC clones in androgen response and response to ARTA. Parental cells could only be stimulated to grow at 10^-10^ and 10^-9^ M R1881, and this stimulus could be completely inhibited by 1 μM ARTA for the 10^-10^ M R1881. In contrast, the CRPC lines showed no significant growth difference between the various conditions and had no response to additional androgens or antiandrogen therapy, suggesting that LAPC4 CRPC clones all developed an AR-independent phenotype, not anymore relying on AR signaling for cell growth.

Importantly, and in contrast to our previous findings in PC346C cells treated with FLU (29), we did not observe any growth induction by the specific antiandrogens used for selection in any of the CRPC clones tested, suggesting the absence of mutations in the ligand-binding domain (LDB) of the AR that would turn these antagonists into agonists.

### Differential Expression of Androgen Receptor and Drivers of Castration Resistance Between Parental Models Identifies WNT Signaling and EMT as Having Increased Expression in an Innate Castration-Resistant Model

**Figure 3:**
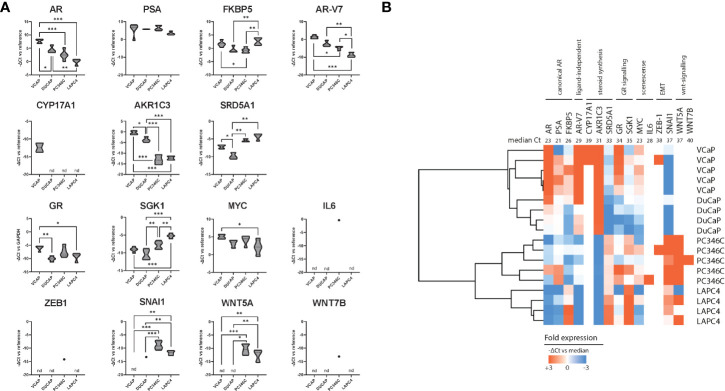
Summary of qPCR analysis of parental cell lines. **(A)** Relative expression of castration resistance-associated genes in parental samples. Summarized qPCR results for parental samples of VCaP (n = 5), DuCaP (n = 4), PC346C (n = 5), and LAPC4 (n = 4). Expression levels are -ΔCt relative to GAPDH and PBGD, meaning that every ΔCt represents a 2-fold difference. * p < 0.05, ** p < 0.01, *** p < 0.001 for Tukey *post-hoc* test. **(B)** Heatmap of clusters identified by differential gene expression of castration resistance-associated genes. Colors indicate -ΔCt vs. median, with red showing a higher expression and blue showing a lower expression. Numbers in the top row indicate median Ct for detection per gene. nd is not detected.

We subsequently tested human mRNA expression of 15 genes commonly associated with castration resistance as compared to the human GAPDH and PBGD housekeeping genes (**Table 1**). We first compared expression levels between individual samples from different passages of parental cell lines to further elucidate *a priori* presence of resistance mechanisms (**Figure 3A**) and account for natural variance in expression levels over time. We found that, in general, the expression of these markers was similar between different passages/samples from the same cell line. Taking into account that every ΔCt (y-axis) means a 2-fold difference, differential expression between the 4 cell lines was found for AR, FKBP5, AR-V7, AKR1C3, SRD5A1, GR, SGK1, SNAI1, and WNT5A. As has been reported previously (44), the expression of AR was highest in VCaP and DuCaP. The expression of PSA was comparable for all parental cell lines. The expression of AR-V7 was highest in VCaP and lowest in LAPC4. The steroidogenic enzymes CYP17A1 and AKR1C3 were highest in VCaP, with CYP17A1 being undetectable in the other models. SRD5A1 was highest in PC346C and LAPC4. GR expression was lowest in DuCaP. GR-target gene SGK1 expression was highest in PC346C and LAPC4. All models expressed similar levels of MYC and no IL6. The expression of the EMT marker SNAI1 was highest in PC346C. The expression of WNT5A was only detectable in PC346C and LAPC4. Clustering expression levels of these markers showed a clear discrimination between the four cell lines, with the closest relation between VCaP and DuCaP, which are derived from different metastases from the same patient (**Figure 3B**).

### Expression Profile of AR, AR-V, GR, Steroidogenesis, and WNT5A in CRPC Clones

Next, we analyzed the differential expression of these 15 genes between CRPC derivatives and parental samples grouped per original cell line to elucidate resistance mechanisms per cell line.

In DuCaP and PC34C, CRPC derivatives showed increased expression of AR, while the expression of AR-V7 was enhanced in CRPC sublines of VCaP, DuCaP, and PC346C compared to parental expression levels (**Supplementary Figures S3****–****S5**). In line with the loss of AR responsiveness, the expression of AR, AR-V7, and AR target genes PSA and FKBP was lower in CRPC sublines of LAPC4 (**Supplementary Figure S6**). Of note, when the expression of AR-V7 was normalized to the expression of AR wt, we found an elevated expression of AR-V7 in both VCaP and PC346C CRPC sublines resistant to BIC and FLU in case of VCaP (**Supplementary Figure S7**). In DuCaP, AR-V7 levels did not increase relative to AR, suggesting that the differential expression of AR variants upon ADT/ARTA may occur but not in every tumor *per se*.

Steroidogenic enzymes CYP17A1, AKR1C3, and SRD5A1 were differentially expressed between CRPC derivatives and parental samples in VCaP and DuCaP but not in PC346C or LAPC4. In contrast, the expression of GR increased in CRPC sublines of PC346C and LAPC4 but not in VCaP or DuCaP. Markers for WNT signaling remained undetectable in VCaP and DuCaP while showing increased expression in CRPC derivatives of PC346C and LAPC4.

**Figure 4:**
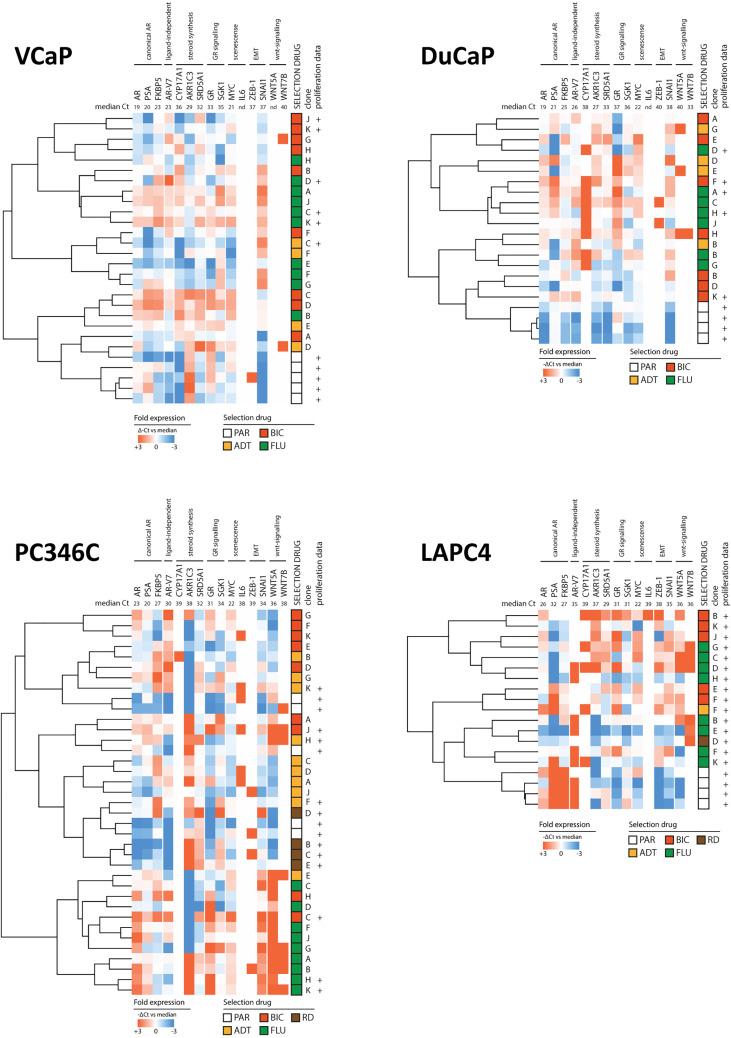
Heatmap of clusters identified by differential gene expression of the 15-gene signature between parental cell lines and ARTA-resistant lines. Clockwise from top left: VCaP, DuCaP, LAPC4, and PC346C. Note that in VCaP, DuCaP, and LAPC, parental samples all have a distinct distance from resistant samples, whereas in PC346C, parental samples mix with resistant samples. In all 4 backgrounds, different types of ARTA did not lead to the rise of a specific cluster, except for OH-flutamide-resistant clones in PC346C. Colors indicate -ΔCt vs. median, with red showing a higher expression and blue showing a lower expression. Numbers in the top row indicate median Ct for detection per gene.

To elucidate whether specific types of ARTA would generate a single phenotype, we clustered the expression levels of the 15 resistance-associated genes of CRPC sublines and parental samples (**Figure 4**). We found that overall, parental samples grouped together in VCaP, DuCaP, and LAPC4 but mixed with CRPC samples in PC346C. In VCaP, DuCaP, and LAPC, no obvious ARTA-specific grouping was observed, while in PC346C, the greatest distance was found between FLU-resistant sublines and the other samples.

## Discussion

In this study, we describe the creation of a total of 88 castration-resistant cell lines by long-term culture from 4 AR-expressing PC models: VCaP, DuCaP, PC346C, and LAPC4. The success rate for the creation of CRPC sublines by culturing in charcoal-stripped medium (ADT condition) or ARTA selection (ADT + BIC, FLU, or RD162) was primarily defined by the cell line origin. PC346C was most successful with the establishment of CRPC sublines in all attempts but one. This parallels our previous reports of PC346C also acquiring resistance to abiraterone, enzalutamide, and docetaxel (45, 46). In VCaP, DuCaP, and LAPC4, the chance of success was defined by the culture condition, with enhanced formation of CRPC sublines in the presence of BIC and FLU. We found profound differences of androgen response of CRPC derivatives. VCaP and DuCaP CRPC sublines showed hypersensitivity to androgens, and PC346C showed increased androgen-independent growth while maintaining androgen response at similar levels of hormone as parental samples. In contrast, LAPC4 sublines showed a complete loss of androgen responsiveness.

Although long-term culture may alter cell line behavior *via* random mutations due to epigenetic changes and chromosomal instability (47), CRPC sublines from a single origin all responded surprisingly similar. This suggests that the cell line origin was the main determinant of the castration-resistant phenotype, as opposed to random effects. This is an important observation: although the generation of castration-resistant derivatives may vary per type of ARTA, the subsequent behavior does not seem to differ by type of ARTA but rather by original cell line background. This parallels a recent report that in ARTA-naive LNCaP, C4-2B, and LAPC4, the immediate response to ARTA was cell line- and not ARTA-specific (48).

In contrast with our previous experience with PC346C (29), showing FLU-induced selection of both AR-overexpressing cells (PC346C-FLU1) and AR-mutant cells (PC346CFLU2), we found only overexpression of AR in the currently generated ARTA-resistant clones. However, none of the sublines showed stimulated cell growth by the respective selection drug (**Figure 2**), indicative that activating mutations in the LBD were not the main driver of resistance in these sublines. The enhanced survival of CRPC culture attempts with ARTA BIC and FLU over ADT alone or RD162 mimics the minimal added value of adding BIC or FLU to ADT in previous clinical trials comparing ADT to MAB (4, 5). Moreover, the reduced formation of CRPC sublines in the presence of RD162 mimics the current clinical trials that have demonstrated superiority of adding apalutamide or enzalutamide to ADT in metastatic hormone-sensitive prostate cancer (mHSPC) (12, 18).

In VCaP and DuCaP, hypersensitivity to androgens leads to growth stimulation in the presence of minute androgen levels and growth inhibition at higher levels of androgens. The potential of this paradoxal mechanism has been observed in the clinic as a PSA response after withdrawal of BIC and FLU (49) and is the basis for bipolar androgen therapy, which has shown promising results in CRCP patients both before and after enzalutamide treatment (50, 51).

Furthermore, both VCaP and DuCaP demonstrated the highest levels of AR and markers for steroid synthesis at baseline. In CRPC sublines of VCaP and DuCaP, the expression of AR, AR variants, and CYP17A1 increased, suggesting that these models remain AR-driven. Moreover, in BIC-resistant PC346C, but not FLU-resistant PC346C, the relative expression of AR-V7 also increased. Potentially, these findings may point toward (weaker) antiandrogens leading to increased expression of variants and steroidogenic enzymes and ultimately resistance. It is likely that the higher antiandrogenic potency of RD162 over BIC (10, 16) blocks the AR more potently, leading to the demise of the persistently AR-dependent VCaP and DuCaP. Alternatively, other mechanisms like the upregulation of UDP-glucuronosyltransferase enzymes by BIC and FLU, as reported in LAPC4 (52), may lead to ligand-independent AR activation *via* activation of kinases (53). This AR activation may in turn still be blocked by later-generation antiandrogens, as these inhibit also AR translocation to the nucleus (46), preventing target gene activation in ways other than blocking the LDB of the AR.

Parental PC346C and LAPC4 demonstrated the lowest levels of AR and enzymes for the steroid synthesis of CYP17A1 and AKR1C3. In both models, the CRPC sublines all showed enhanced AR-independent growth and unchanged to undetectable levels of enzymes for steroidogenesis. This suggests little to minimal *a priori* dependence on canonical AR signaling, which is further enhanced by selection with ADT and ARTA.

GR expression has been reported as a driver of enzalutamide resistance in LNCaP xenografts (32) and also in abiraterone-resistant VCaP (54). This observation was confirmed in patients with GR-positive metastases, who showed little response to enzalutamide (32). In this context, the AR-negative/GR-positive phenotype of all LAPC4 CRPC clones in our study is highly interesting. In contrast to LNCaP used in the report by Arora et al. (32), we found no restoration of PSA in LAPC4 CRPC clones by qPCR. In PC346C, ARTA-resistant sublines showed an increased expression of GR, with similar PSA mRNA levels compared to parental cells. However, both models demonstrated substantial AR-independent growth *in vitro* that was unaffected by RD162. This may indicate that enhanced PSA expression may well be regardless of GR status. Since the AR was suggested to be a negative transcription regulator of GR, further studies are needed to define if the GR is an active driver of resistance or a bystander effect of altered AR dependency.

PC346C—the most resilient model in terms of ability to form CRPC sublines relative to the other three—was the only cell line that had detectable and substantial expression of SNAI1 and WNT5A at baseline, with elevated expression levels in CRPC derivatives. While undetectable at baseline, SNAI1 expression became detectable in VCaP and DuCaP at progression to CRPC. In LAPC4, CRPC sublines expressed both ZEB-1 and SNAI1, while also WNT7B expression was enhanced. This warrants further interrogation of EMT and WNT signaling markers in the HSPC population if these may predict a subsequent treatment response.

## Conclusion

Taken together, an important observation can be made: in our well-defined setup, the potency to become castration-resistant and the mechanism of castration resistance seem to be already defined before full resistance has occurred. If true for complex PCa biology in patients, response to hormone therapy could be comprehended up front and may identify patients who need aggressive treatment early on based on biological behavior rather than the currently surrogate measure of aggressiveness: extent of disease at diagnosis (55, 56).

## Data Availability Statement

The raw data supporting the conclusions of this article will be made available by the authors without undue reservation.

## Author Contributions

JM, WW, and GJ contributed to conception and design of the study. JM, WT, SE, AJ-A, ND, and AR performed the experiments and collected the data. SE, AJ-A, ND, and AR organized the database. JM performed the statistical analysis. JM, WW, and GJ wrote the article. All authors contributed to article revision and read and approved the submitted version.

## Funding

This project was supported by TI-Pharma grant T3-107.

## Conflict of Interest

The authors declare that the research was conducted in the absence of any commercial or financial relationships that could be construed as a potential conflict of interest.

## Publisher’s Note

All claims expressed in this article are solely those of the authors and do not necessarily represent those of their affiliated organizations, or those of the publisher, the editors and the reviewers. Any product that may be evaluated in this article, or claim that may be made by its manufacturer, is not guaranteed or endorsed by the publisher.

## Abbreviations

HSPC, hormone-sensitive prostate cancer; MAB, maximal androgen blockade; PCa, prostate cancer; mPC, metastatic prostate cancer; CRPC, castration-resistant prostate cancer; AR, androgen receptor; AR-V7, androgen receptor splice variant 7; GR, glucocorticoid receptor; PSA, prostate-specific antigen; FKBP5, FK506-binding protein 5; SGK1, serum and glucocorticoid-regulated kinase 1; LBD, ligand-binding domain.
