# Supplementary figures and images for: Cell Line Characteristics Predict Subsequent Resistance to Androgen Receptor-Targeted Agents (ARTA) in Preclinical Models of Prostate Cancer
Source: Front Oncol. 2022 Jun 13;12:877613. doi: 10.3389/fonc.2022.877613 (PMC9234122; doi:10.3389/fonc.2022.877613)

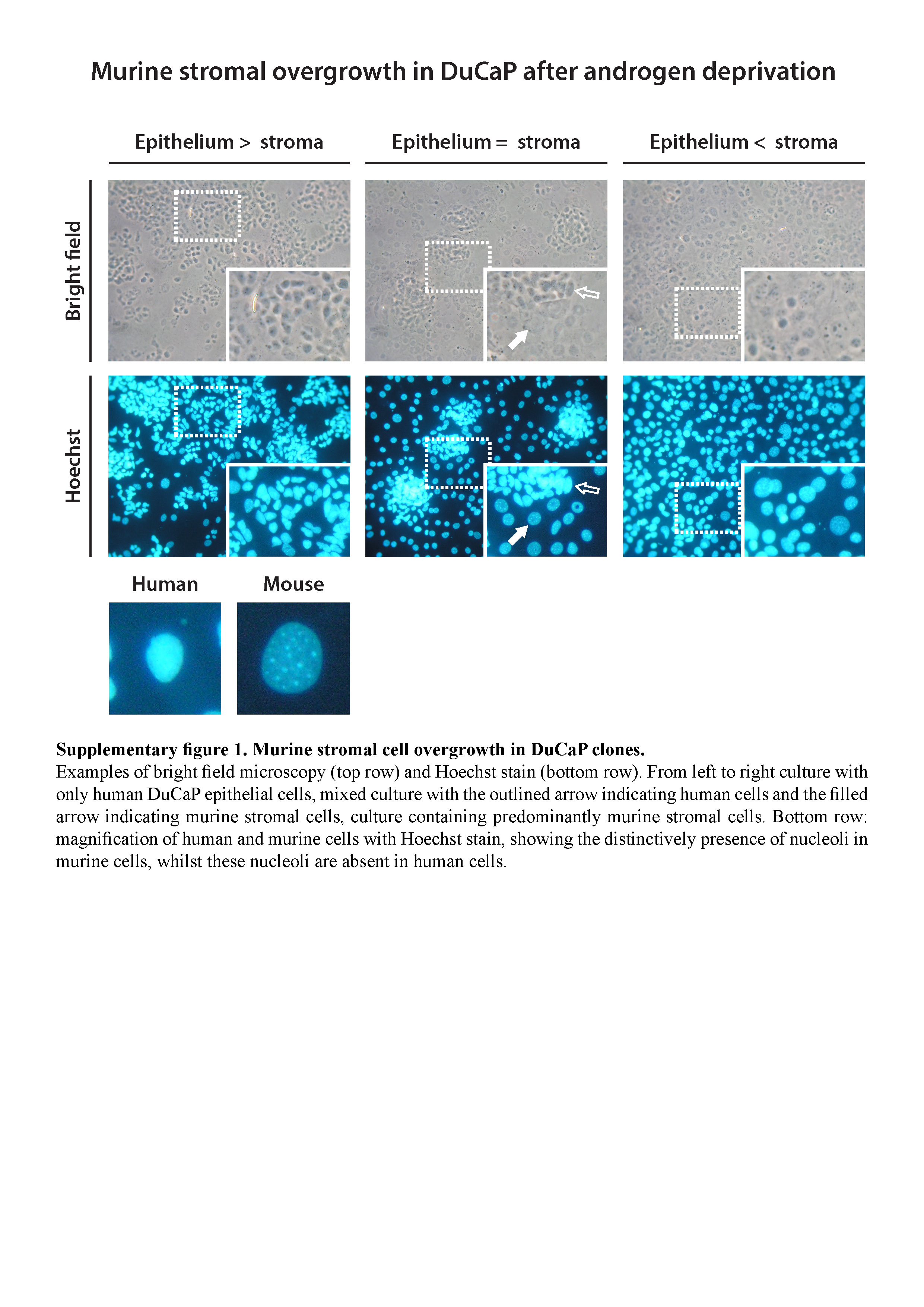

Supplement: Supplementary file 1 [file Image_1.jpeg]

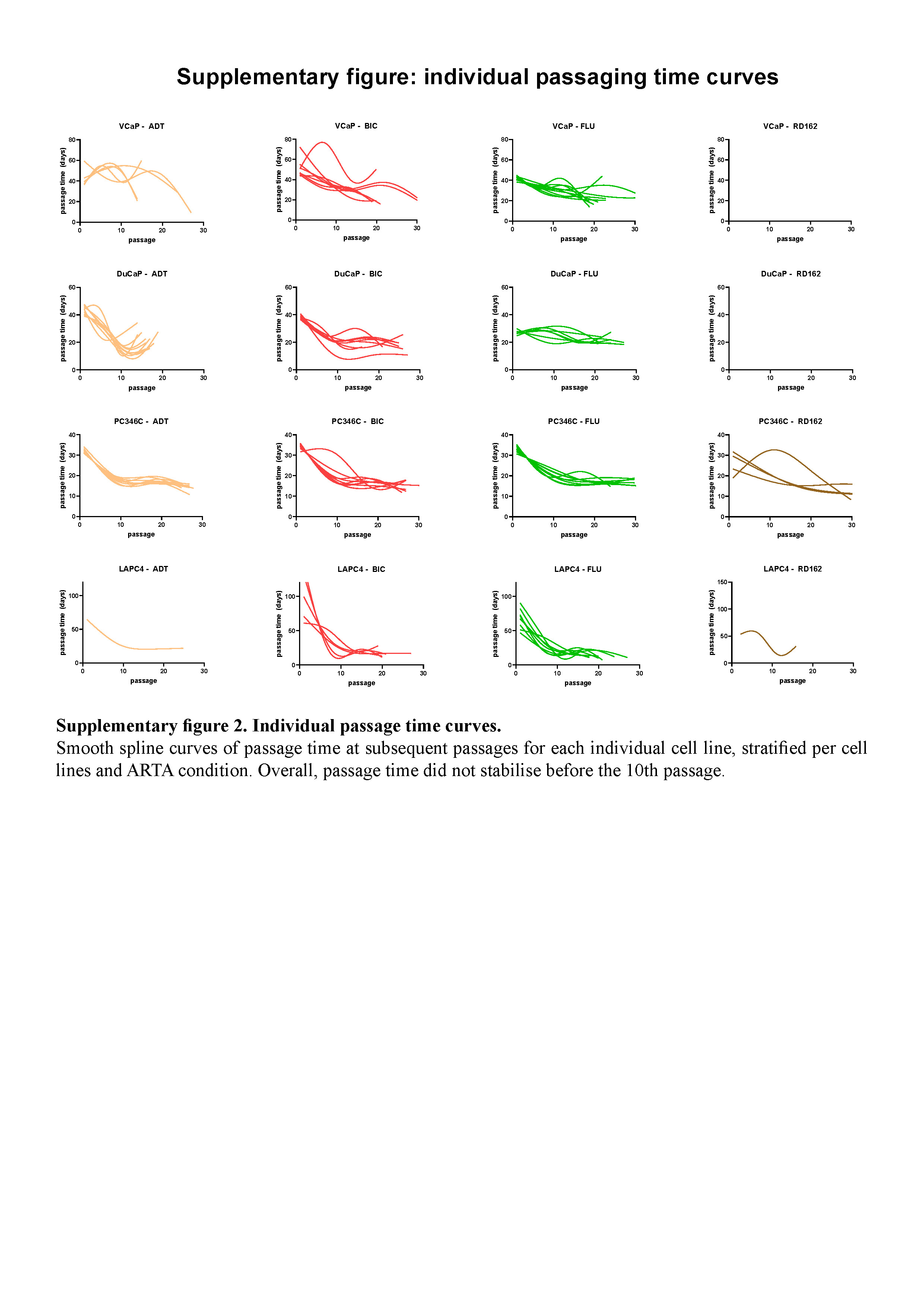

Supplement: Supplementary file 2 [file Image_2.jpeg]

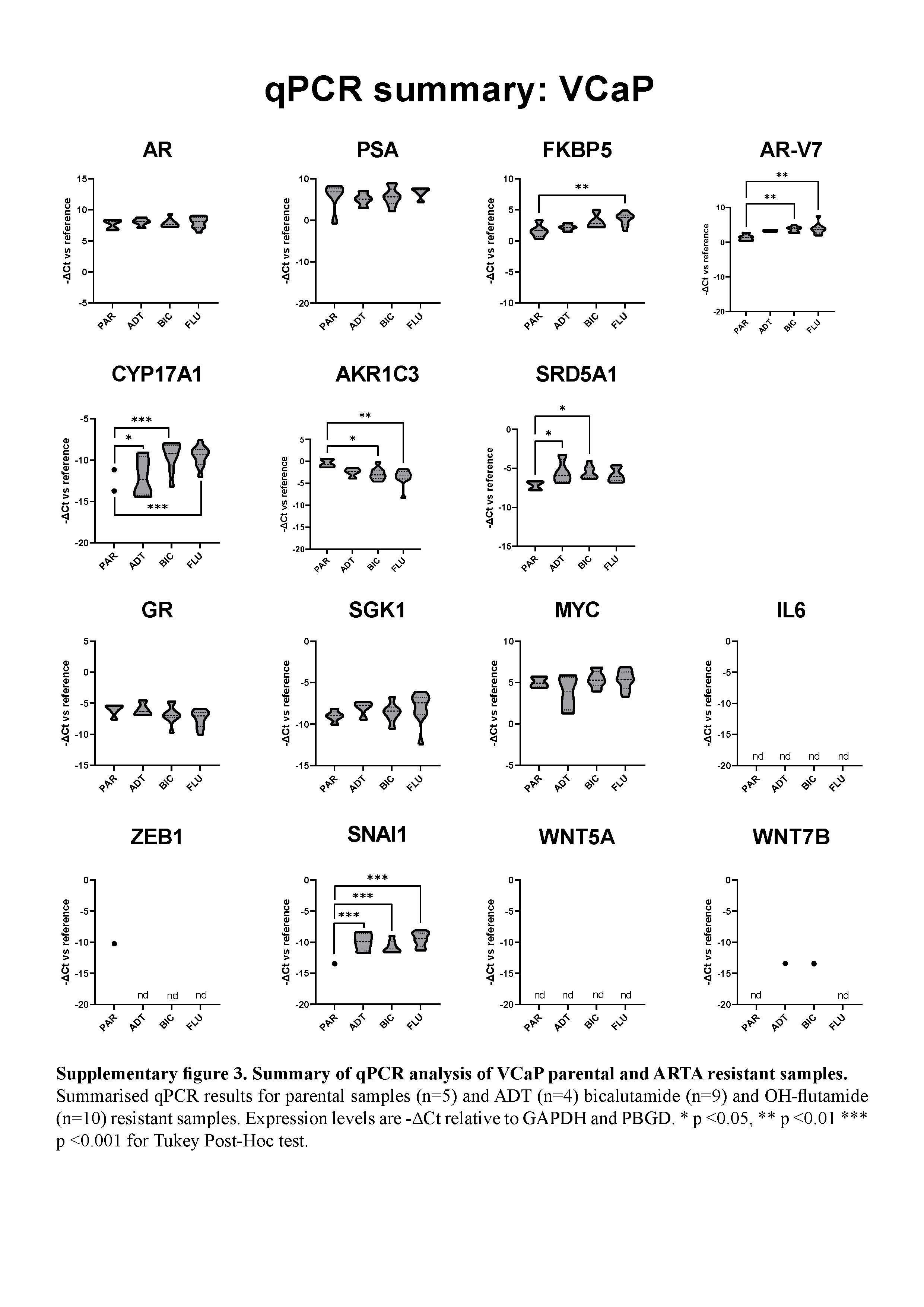

Supplement: Supplementary file 3 [file Image_3.jpeg]

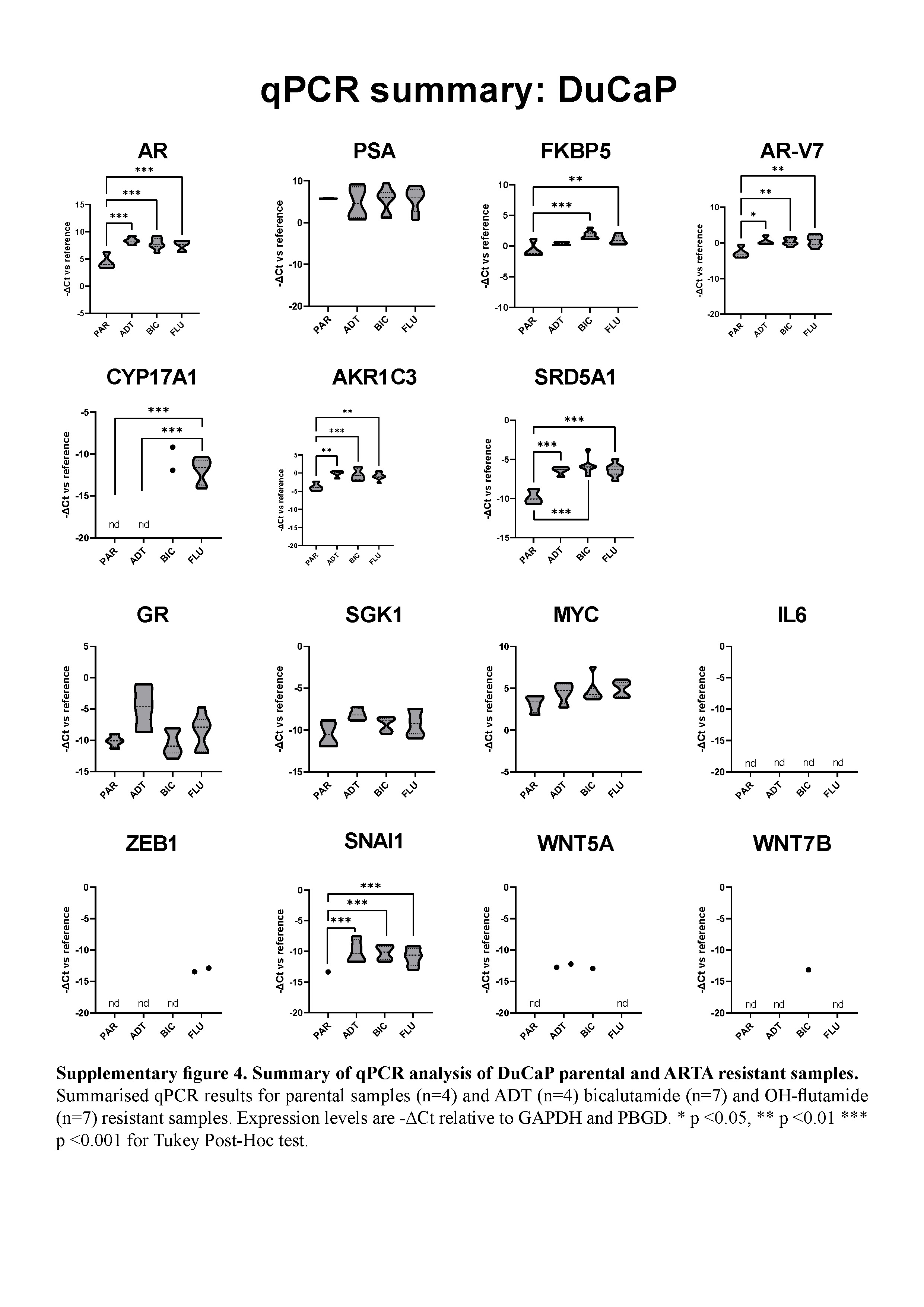

Supplement: Supplementary file 4 [file Image_4.jpeg]

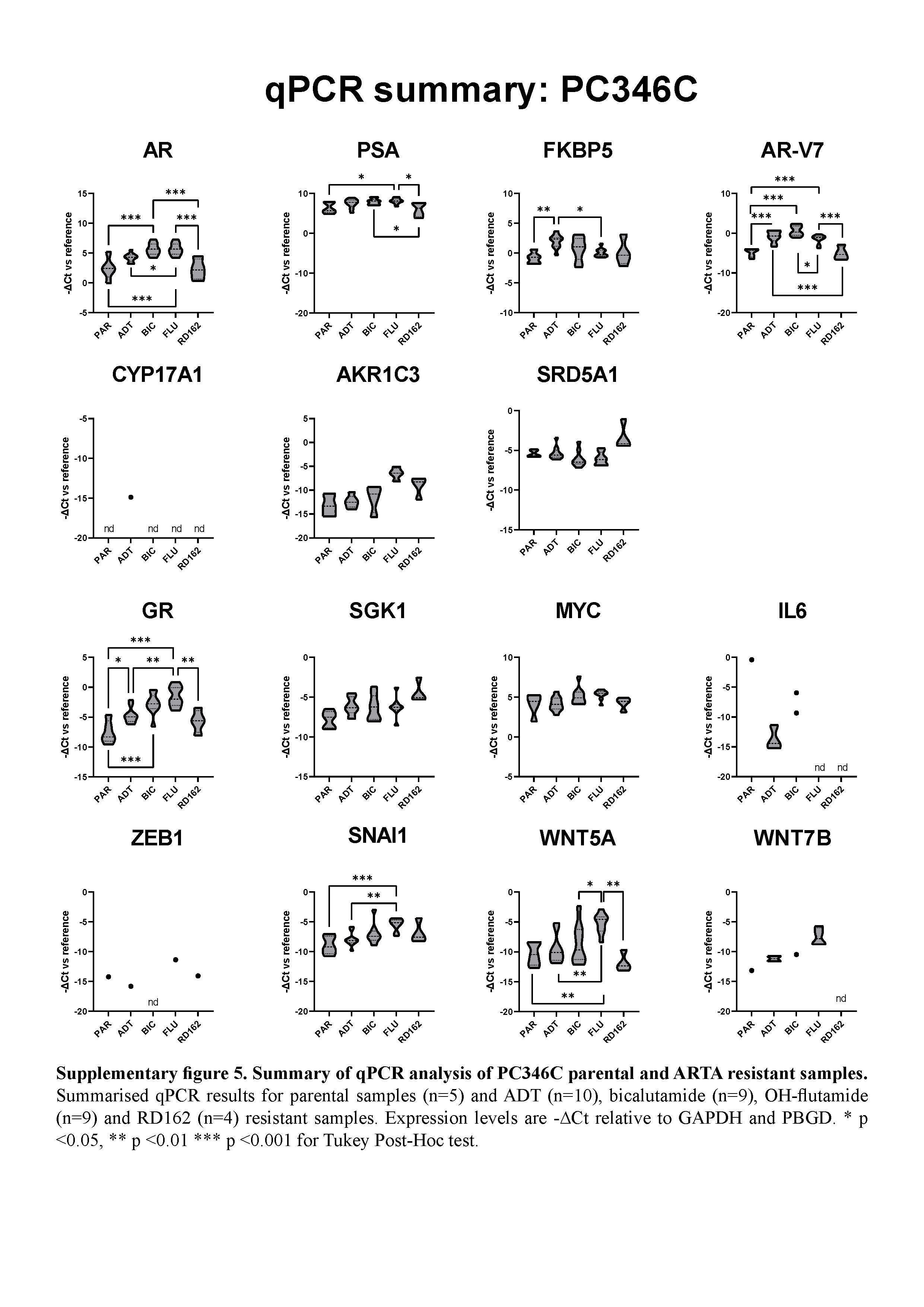

Supplement: Supplementary file 5 [file Image_5.jpeg]

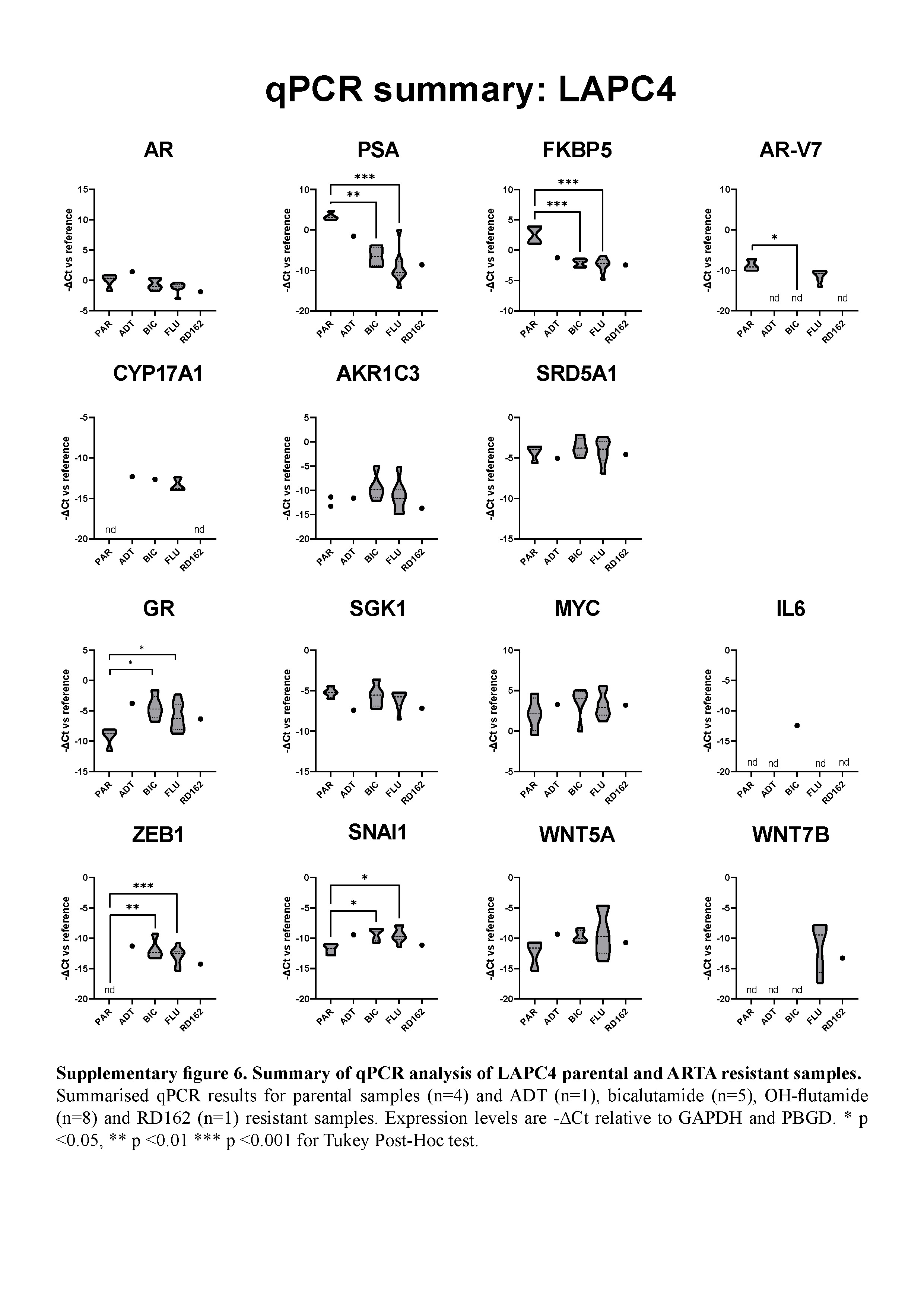

Supplement: Supplementary file 6 [file Image_6.jpeg]

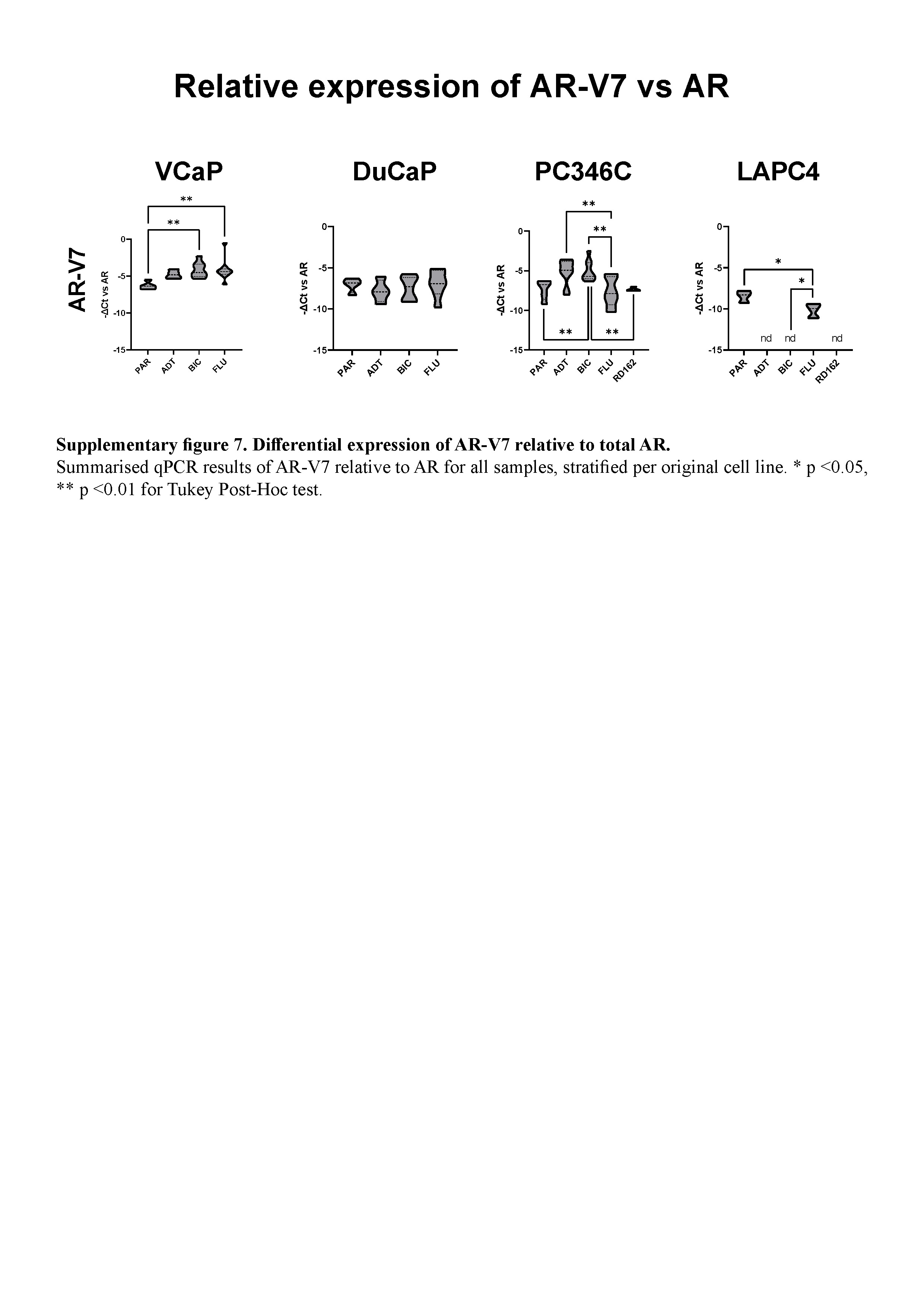

Supplement: Supplementary file 7 [file Image_7.jpeg]
